# Impact of overestimation of fractional flow reserve by adenosine on anatomical–functional mismatch

**DOI:** 10.1038/s41598-022-19330-1

**Published:** 2022-09-02

**Authors:** Hidenari Matsumoto, Ryota Masaki, Satoshi Higuchi, Hideaki Tanaka, Seita Kondo, Hiroaki Tsujita, Toshiro Shinke

**Affiliations:** Division of Cardiology, Showa University School of Medicine, 1-5-8 Hatanodai, Shinagawa-ku, Tokyo, 142-8555 Japan

**Keywords:** Cardiology, Interventional cardiology

## Abstract

Adenosine occasionally results in overestimation of fractional flow reserve (FFR) values, compared with other hyperemic stimuli. We aimed to elucidate the association of overestimation of FFR by adenosine with anatomically significant but functionally non-significant lesions (anatomical–functional mismatch) and its influence on reclassification of functional significance. Distal-to-aortic pressure ratio (Pd/Pa) was measured using adenosine (Pd/Pa_ADN_) and papaverine (Pd/Pa_PAP_) in 326 patients (326 vessels). The overestimation of FFR was calculated as Pd/Pa_ADN_–Pd/Pa_PAP_. The anatomical–functional mismatch was defined as diameter stenosis > 50% and Pd/Pa_ADN_ > 0.80. Reclassification was indicated by Pd/Pa_ADN_ > 0.80 and Pd/Pa_PAP_ ≤ 0.80. The mismatch (n = 72) had a greater overestimation of FFR than the non-mismatch (n = 99): median 0.02 (interquartile range 0.01–0.05) versus 0.01 (0.00–0.04), *p* = 0.014. Multivariable analysis identified the overestimation of FFR (*p* = 0.003), minimal luminal diameter (*p* = 0.001), and non-left anterior descending artery (LAD) location (*p* < 0.001) as determinants of the mismatch. Reclassification was indicated in 29% of the mismatch and was more frequent in the LAD than in the non-LAD (52% vs. 20%, *p* = 0.005). The overestimation of FFR is an independent determinant of anatomical−functional mismatch. Anatomical−functional mismatch, specifically in the LAD, may suggest a false-negative result.

## Introduction

Diameter stenosis (DS) of > 50% on invasive coronary angiography is deemed anatomically significant^1^. Coronary angiography alone, however, has limited ability to differentiate functionally significant lesions^2–7^. Pressure wire-derived fractional flow reserve (FFR) has emerged as the gold standard for assessing the functional impact of stenotic lesions, and a vessel with an FFR value ≤ 0.80 is considered to have functionally significant stenosis^2,8–11^. It is well known that the anatomical and functional significance of coronary lesions does not always match^2–4^. Previous studies have identified clinical and pathophysiological factors associated with anatomically significant but functionally non-significant lesions (anatomical–functional mismatch)^3–7,12^.

The principle of FFR is based on the premise that coronary pressure is linearly related to myocardial blood flow under maximal hyperemia^9,13^. If maximal hyperemia is not induced, an FFR value becomes erroneously higher than the true FFR value. This overestimation of FFR underestimates the severity of ischemia and poses a risk of leaving an ischemic lesion untreated^14^. Intravenous adenosine, used most widely for hyperemia induction^13,15^, occasionally fails to induce maximal hyperemia when compared with other hyperemic stimuli, especially if the subject has consumed caffeine, a competitive antagonist of the target receptor of adenosine^16–20^. Although an overestimation of FFR by adenosine may cause an anatomical–functional mismatch (DS > 50% and FFR > 0.80), the association between the overestimation of FFR by adenosine and anatomical–functional mismatch has not been fully explored. Previous studies on anatomical–functional mismatch have used only adenosine to produce hyperemia, thereby precluding assessment of adenosine’s role in the mismatch^3–6,12^. If the anatomical–functional mismatch is associated with the overestimation of FFR by adenosine, vessels having the mismatch with adenosine may show functional significance when using another stimulus.

Papaverine induces maximal hyperemia most reliably by directly relaxing the vascular smooth muscle, without involving the adenosine A_2a_ receptors^16–19,21^. This study compared distal-to-aortic pressure ratio (Pd/Pa) associated with adenosine (Pd/Pa_ADN_) and Pd/Pa associated with papaverine (Pd/Pa_PAP_), as a reference standard. We sought to determine whether (1) the overestimation of FFR by adenosine is associated with the discordance between the anatomical and functional significance and (2) functional significance in such lesions is reclassified when papaverine is used as the indicator.

## Methods

### Study patients

This study retrospectively analyzed the data of 365 consecutive patients with chronic coronary syndrome undergoing clinically indicated coronary angiography and an FFR assessment for coronary stenosis of 30–90% based on visual estimations during angiography. If FFR was measured in two or more vessels in a patient, only the first vessel was included in this study. All patients were asked to abstain from food and beverages for > 3 h before the catheterization. Periods for caffeine abstinence were left to the referring physicians’ discretion. This study excluded vessels with lesions on the coronary ostium, severe arrhythmia (e.g. atrial fibrillation or frequent ectopic beats), a prior coronary artery bypass graft, significant valvular disease, or any contraindications for adenosine or papaverine, as well as patients taking theophylline-containing medications. Patients with insufficient pressure data quality, including inadequate waveform tracings and signal drift more than ± 0.03 after the pullback of the pressure wire, were also excluded from the analysis.

The coronary physiology assessment was performed as part of the routine diagnostic coronary angiography procedures for clinical purposes. All methods were performed in accordance with the relevant guidelines and regulations. Written informed consent for the invasive physiology assessment was obtained from all of the patients before the procedure. The Institutional Review Board approved this retrospective study and waived the requirement to obtain patient approval and written informed consent for the review of patient data and medical records (reference #3234/ Showa University School of Medicine; 31 August, 2021).

### Fractional flow reserve measurements

Diagnostic coronary angiography was performed in a standard manner. Thereafter, the FFR was measured using a 5- or 6-F guiding catheter without side holes and a commercially available FFR system (Philips Volcano or Abbott Vascular) in accordance with standardized procedures^13^. After administration of intracoronary isosorbide dinitrate, distal coronary pressure and aortic pressure were simultaneously measured at baseline and during hyperemia. The guiding catheter was disengaged from the ostium during FFR measurements, and special care was taken not to alter the wire position.

Adenosine was continuously given via a femoral vein or a large forearm vein at a dose of 140 μg/kg/min for more than 150 s^9,13,15,22^. When Pd/Pa values were not stable during adenosine infusion, adenosine was continued for more than 180 s. Papaverine was used as the last agent to obtain a reliable pull-back curve, as it induces hyperemia with minimal variations in Pd/Pa^23^. After confirming that Pd/Pa values had returned to the baseline level, with an interval of ≥ 5 min, intracoronary papaverine (8–10 mg in the right coronary artery or 12–15 mg in the left coronary artery) was given through the coronary catheter, followed by 5 ml of saline^9,13^. Approximately 20 s after the papaverine injection, an FFR pullback recording was performed manually, and the presence of pressure-wire drift was checked.

### Data analysis

#### Fractional flow reserve

Experienced observers blinded to patients’ coronary angiography results and clinical data manually reviewed the pressure recordings. Pd/Pa_ADN_ was determined during the steady-state hyperemic plateau phase > 60 s after the initiation of adenosine and > 15 s after the transition to hyperemia^19,24^. The lowest Pd/Pa values on a beat-to-beat basis for adenosine and papaverine were regarded as Pd/Pa_ADN_ and Pd/Pa_PAP_, respectively^16–19,25^. The overestimation of FFR by adenosine was defined as Pd/Pa_ADN_ – Pd/Pa_PAP_^19^.

#### Coronary angiography

Quantitative coronary angiography was performed with a commercially available system (CAAS Workstation version 7.5, Pie Medical Imaging) by independent investigators unaware of patients’ FFR results or clinical data. Reference diameter, minimum lumen diameter, and lesion length were measured using the external diameter of the catheter as a scaling device, and DS was calculated. Proximal location was defined as Syntax segments 1, 5, 6, and 11^26^. The anatomical–functional mismatch was defined as DS > 50% and Pd/Pa_ADN_ > 0.80, and the reverse mismatch was defined as DS ≤ 50% and Pd/Pa_ADN_ ≤ 0.80.

### Reclassification of functional significance

Reclassification of functional significance was indicated by FFR_ADN_ > 0.80 and FFR_PAP_ ≤ 0.80 (false-negative by adenosine), and reverse reclassification as FFR_ADN_ ≤ 0.80 and FFR_PAP_ > 0.80 (false-positive by adenosine).

### Statistical analysis

Continuous variables were presented as medians with interquartile ranges (IQRs). Categorical variables were expressed as frequencies with percentages. Between-group comparisons were made with unpaired-sample t-test or Mann–Whitney U test for quantitative variables and with the χ^2^ test or Fisher’s exact test for categorical variables, as appropriate. The correlation between DS and Pd/Pa_ADN_ was assessed with Spearman’s rank correlation coefficient. Multivariable logistic regression analysis was used to determine factors associated with the anatomical–functional mismatch and the reverse mismatch. Clinical, angiographic, and hemodynamic parameters with a univariable association of *p* < 0.10 were entered into the multivariable model. Results were presented as odds ratios (ORs) and 95% confidence intervals (CIs). The numbers of the mismatch in vessels with DS > 50% and the reverse mismatch in vessels with DS ≤ 50% were compared between adenosine and papaverine using the McNemar test. Statistical analyses were performed using JMP**®** Pro, version 16.0.0. (SAS Institute Inc., Cary, NC, USA). A *p* value of < 0.05 was considered statistically significant.

## Results

### Patient and lesion characteristics

Of the 365 patients, 39 were excluded due to sensor drift (n = 13), insufficient waveform tracings (n = 10), side effects of adenosine (n = 8) or papaverine (n = 2), or difficulty in advancing the pressure wire far enough distal from the index lesion (n = 6), leaving 326 patients (326 vessels) in the analysis. Table 1 summarizes the patient and lesion characteristics.

**Table 1:** Patient and lesion characteristics.

No. of patients	326
Age, yrs	72 (65–78)
Male, n (%)	252 (77%)
Body mass index, kg/m^2^	23.8 (21.7–25.9)
Hypertension, n (%)	232 (71%)
Diabetes mellitus, n (%)	133 (41%)
Dyslipidemia, n (%)	235 (72%)
Target vessel (non-LAD), n (%)	135 (41%)
Proximal lesion*, n (%)	121 (37%)
Multivessel disease, n (%)	179 (55%)
**Quantitative coronary angiography**	
Reference diameter, mm	2.8 (2.4–3.3)
Minimal luminal diameter, mm	1.4 (1.1–1.7)
DS, %	50.6 (42.4–57.5)
Lesion length, mm	11.5 (8.1–16.4)
**Hemodynamic parameters**	
Heart rate at baseline, beats/min	67 (61–74)
Pa at baseline, mmHg	91 (82–101)
Pd/Pa ratio at baseline	0.93 (0.88–0.96)
Pd/Pa_ADN_	0.79 (0.73–0.86)
Pd/Pa_PAP_	0.77 (0.70–0.84)

Values are expressed as medians (interquartile ranges) or frequencies (percentages).

*Proximal location was defined as Syntax segments 1, 5, 6, and 11.

*DS* diameter stenosis, *LAD* left anterior descending coronary artery, *Pa* aortic pressure, *Pd/Pa* distal-to-aortic pressure ratio, *Pd/Pa*_*ADN*_ distal-to-aortic pressure ratio associated with adenosine, *Pd/Pa*_*ADN*_ distal-to-aortic pressure ratio associated with papaverine.

**Figure 1:**
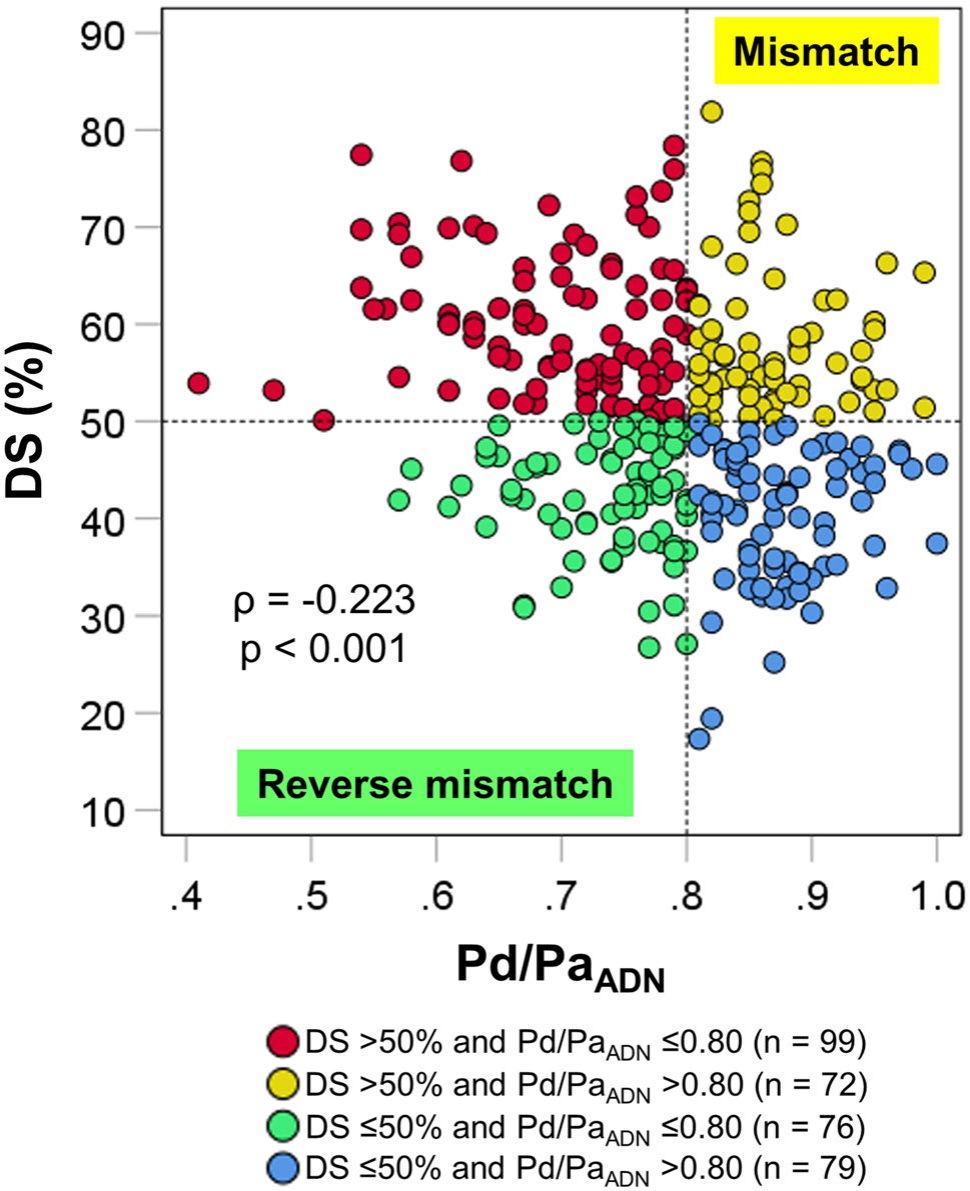
Scatter plot of DS and Pd/Pa_ADN_ The anatomical‒functional mismatch was defined as DS > 50% and Pd/Pa_ADN_ > 0.80, and the reverse mismatch as DS > 50% and Pd/Pa_ADN_ ≤ 0.80. *DS* diameter stenosis, and *Pd/Pa*_*ADN*_ distal-to-aortic pressure ratio associated with adenosine.

The median DS was 50.6% (IQR 42.4–57.5%), and the median Pd/Pa_ADN_ was 0.79 (0.73–0.86). Figure 1 shows a scatter plot of DS and Pd/Pa_ADN_. The relationship between DS and Pd/Pa_ADN_ was modest, with a large scatter (ρ =−0.223, *p* < 0.001). DS > 50% was observed in 171 vessels (52%), and DS ≤ 50% in 155 vessels (48%). Among the vessels with DS > 50%, 72 and 99 vessels demonstrated Pd/Pa_ADN_ > 0.80 and Pd/Pa_ADN_ ≤ 0.80, respectively. Of the vessels with DS ≤ 50%, 76 and 79 vessels showed Pd/Pa_ADN_ ≤ 0.80 and Pd/Pa_ADN_ > 0.80, respectively.

### Anatomically significant vessels

Table 2 compares patient and lesion characteristics in vessels with DS > 50% between the mismatch (Pd/Pa_ADN_ > 0.80) and non-mismatch (Pd/Pa_ADN_ ≤ 0.80) groups. There was no significant difference in age, sex, body size, or coronary risk factors between groups. Concerning lesion characteristics, the mismatch group was associated with a higher frequency of non-LAD location (71% vs. 27%, *p* < 0.001), a greater reference diameter (3.1 mm [IQR 2.6–3.5 mm] vs. 2.7 mm [IQR 2.2–3.2 mm], *p* = 0.001), a larger minimal luminal diameter (1.3 mm [IQR 1.1–1.5 mm] vs. 1.1 mm [IQR 0.9–1.3 mm], p < 0.001), a smaller DS (55.8% [IQR 52.8–61.3%] vs. 59.0% [IQR 53.9–64.9%], *p* = 0.038), and a shorter lesion length (11.2 mm [IQR 7.9–15.8] vs. 13.2 mm [IQR 9.3–20.8], *p* = 0.029) than the non-mismatch group.

**Table 2:** Comparion clinical and lesion characteristics in vessels with DS > 50% between the anatomical–functional mismatch and non-mismatch groups.

	Mismatch Pd/Pa_ADN_ > 0.80 (n = 72)	Non-mismatch Pd/Pa_ADN_ ≤ 0.80 (n = 99)	*p* value
Age, yrs	72 (66–78)	72 (65–78)	0.466
Male, n (%)	55 (76%)	82 (83%)	0.298
Body mass index, kg/m^2^	23.6 (21.7–25.8)	23.8 (22.2–26.4)	0.278
Hypertension, n (%)	47 (65%)	65 (66%)	0.959
Diabetes mellitus, n (%)	29 (40%)	42 (42%)	0.553
Dyslipidemia, n (%)	58 (81%)	76 (76%)	0.779
Target vessel (LAD), n (%)	21 (29%)	72 (73%)	< 0.001
Proximal lesion*, n (%)	19 (26%)	38 (38%)	0.100
Multivessel disease, n (%)	41 (57%)	53 (54%)	0.658
**Quantitative coronary angiography**			
Reference diameter, mm	3.1 (2.6–3.5)	2.7 (2.2–3.2)	0.001
Minimal luminal diameter, mm	1.3 (1.1–1.5)	1.1 (0.9–1.3)	< 0.001
DS, %	55.8 (52.8–61.3)	59.0 (53.9–64.9)	0.038
Lesion length, mm	11.2 (7.9–15.8)	13.2 (9.3–20.8)	0.029
Hemodynamic parameters			
Heart rate at baseline, beats/min	67 (61–75)	68 (62–77)	0.749
Pa at baseline, mmHg	92 (82–106)	90 (82–102)	0.349
Pd/Pa ratio at baseline	0.97 (0.93–0.99)	0.88 (0.84–0.92)	< 0.001
Pd/Pa_ADN_	0.86 (0.82–0.89)	0.72 (0.65–0.77)	N.A
Pd/Pa_PAP_	0.84 (0.80–0.87)	0.69 (0.61–0.76)	N.A

Values are expressed as medians (interquartile ranges) or frequencies (percentages).

The mismatch was defined as DS > 50% and Pd/Pa_ADN_ > 0.80, and the non-mismatch as DS > 50% and Pd/Pa_ADN_ ≤ 0.80.

*Proximal location was defined as Syntax segments 1, 5, 6, and 11.

*DS* diameter stenosis, *LAD* left anterior descending coronary artery, *Pa* aortic pressure, *Pd/Pa* distal-to-aortic pressure ratio, *Pd/Pa*_*ADN*_ distal-to-aortic pressure ratio associated with adenosine, *Pd/Pa*_*ADN*_ distal-to-aortic pressure ratio associated with papaverine.

**Figure 2:**
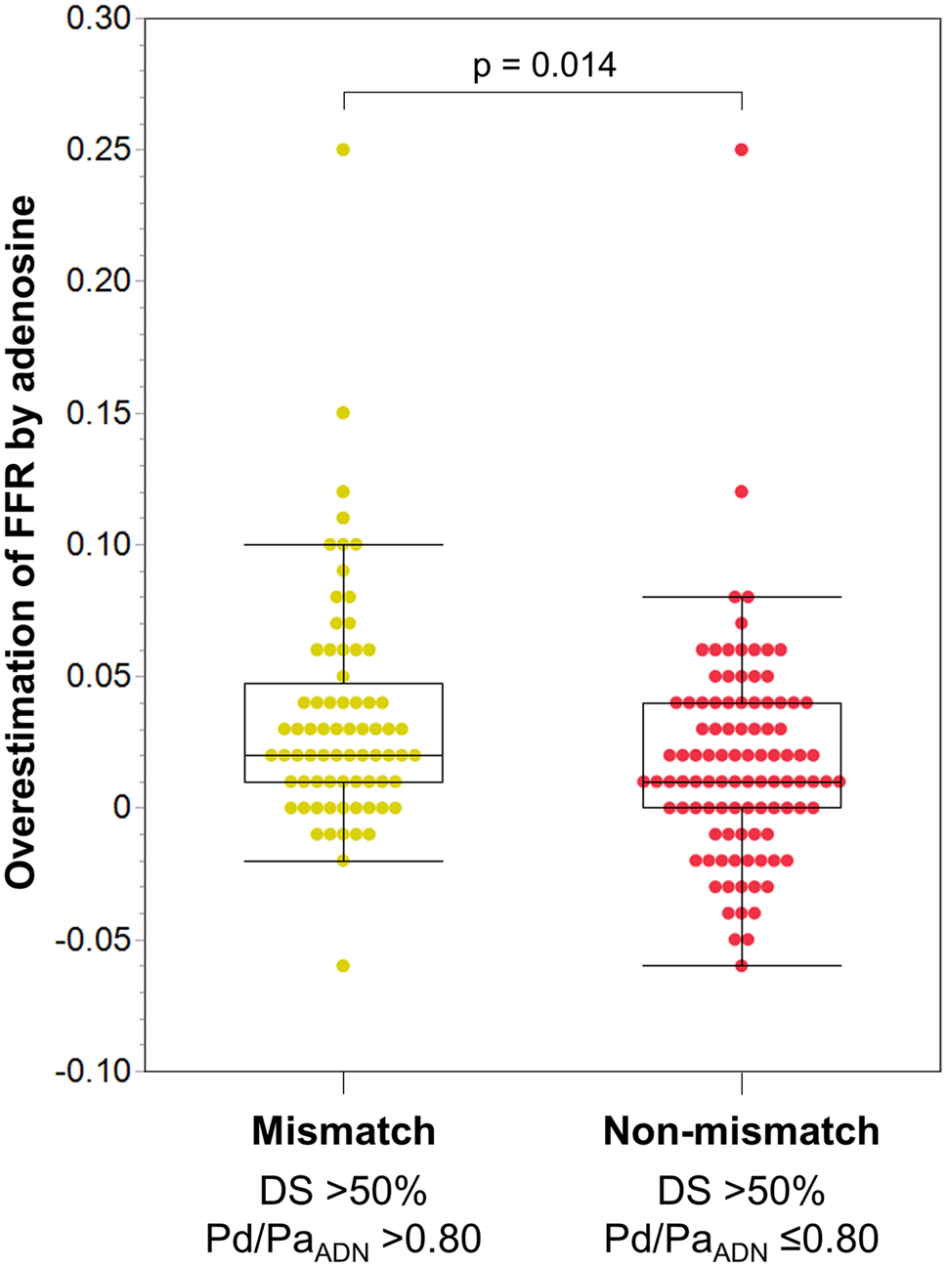
Comparison of overestimation of FFR by adenosine in anatomically significant vessels between the anatomical‒functional mismatch and non-mismatch groups. Distributions of overestimation of FFR by adenosine with box-and-whisker plots are shown. The overestimation of FFR by adenosine was defined as Pd/Pa_ADN_–Pd/Pa_PAP_. *DS* diameter stenosis*, Pd/Pa*_*ADN*_ distal-to-aortic pressure ratio associated with adenosine, and *Pd/Pa*_*ADN*_ distal-to-aortic pressure ratio associated with papaverine.

Pd/Pa_ADN_ values were significantly higher than Pd/Pa_PAP_ values, regardless of the presence of anatomical–functional mismatch (0.78 [IQR 0.70–0.85] vs. 0.76 [IQR 0.68–0.83], *p* < 0.001 for all vessels with DS > 50%; 0.86 [IQR 0.82–0.89] vs. 0.84 [IQR 0.80–0.87], *p* < 0.001 for the mismatch group; and 0.72 [IQR 0.65–0.77] vs. 0.69 [IQR 0.61–0.76], *p* < 0.001 for the non-mismatch group). Figure 2 depicts the distributions of the overestimation of FFR by adenosine in the two groups. The mismatch group had a greater overestimation than the non-mismatch group (0.02 [IQR 0.01–0.05] vs. 0.01 [IQR 0.00–0.04], *p* = 0.014).

Based on the results from the univariable analysis (Suppl. Table S1), we entered the overestimation of FFR (*p* = 0.014), non-LAD location (*p* < 0.001), reference diameter (*p* = 0.004), and minimal luminal diameter (*p* = 0.001) in the multivariable model. As shown in Table 3, multivariable logistic regression analysis identified the overestimation of FFR (OR per 0.01 increase: 1.16 [95% CI: 1.05–1.28], *p* = 0.003), non-LAD location (OR: 9.08 [95% CI: 4.15–19.88], *p* < 0.001), and minimal luminal diameter (OR per 0.1 mm increase: 1.31 [95% CI: 1.11–1.54], *p* = 0.001) as independent factors associated with the anatomical–functional mismatch.

**Table 3:** Association with anatomical-functional mismatch in multivariable analysis.

	OR	95% CI	*p* value
Non-LAD location	9.08	4.15–19.88	< 0.001
Reference diameter (per 0.1 mm increase)	0.97	0.89–1.05	0.427
Minimal luminal diameter (per 0.1 mm increase)	1.31	1.11–1.54	0.001
Pd/Pa_ADN_ – Pd/Pa_PAP_ (per 0.01 increase)	1.16	1.05–1.28	0.003

*CI* confidence interval, *LAD* left anterior descending coronary artery, *OR* odds ratio, *Pd/Pa*_*ADN*_ distal-to-aortic pressure ratio associated with adenosine,* Pd/Pa*_*ADN*_ distal-to-aortic pressure ratio associated with papaverine.

**Figure 3:**
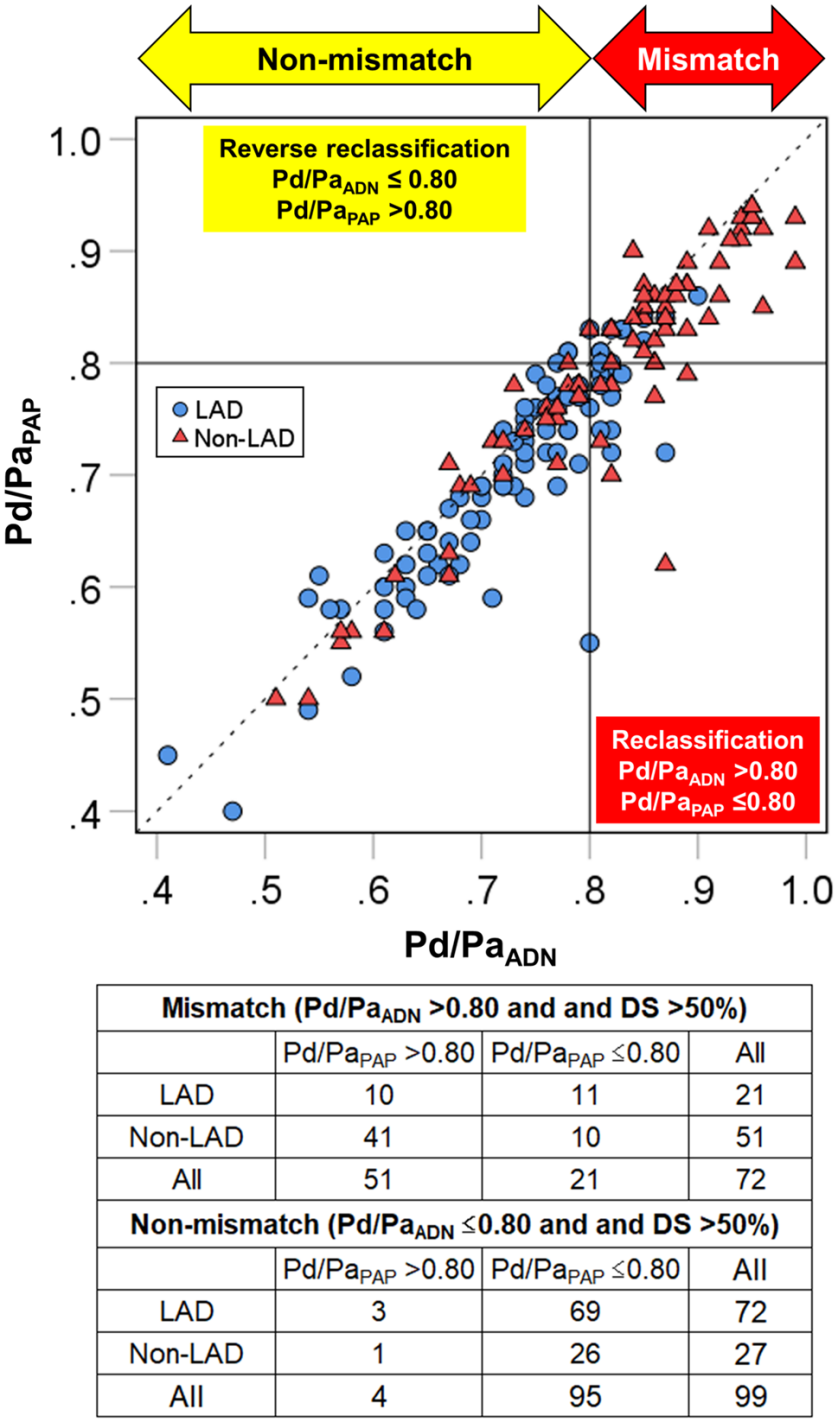
Scatter plot of Pd/Pa_ADN_ and Pd/Pa_PAP_ in vessels with a diameter stenosis of > 50%. The dashed line indicates the line of identity. *LAD* left anterior descending artery, *Pd/Pa*_*ADN*_ distal-to-aortic pressure ratio associated with adenosine, and *Pd/Pa*_*ADN*_ distal-to-aortic pressure ratio associated with papaverine.

Figure 3 displays a scatter plot of Pd/Pa_ADN_ and Pd/Pa_PAP_ in vessels with DS > 50%. Compared to adenosine, papaverine significantly decreased the number of vessels with anatomical–functional mismatch (99 [58%] vs. 116 [68%], *p* = 0.001). In the mismatch group, the reclassification of functional significance by papaverine (Pd/Pa_ADN_ > 0.80 and Pd/Pa_PAP_ ≤ 0.80) was observed in 29% (21/72). Of these, 12 vessels had an overestimation of FFR of ≥ 0.05, and 7 vessels had Pd/Pa_PAP_ values of ≤ 0.75. Reclassification was more frequently observed in LAD lesions than in non-LAD lesions (52% [11/21] vs. 20% [10/51], *p* = 0.005). In the non-mismatch group, reverse reclassification (Pd/Pa_ADN_ ≤ 0.80 and Pd/Pa_PAP_ > 0.80) was observed in only 4% (4/99). In all vessels, Pd/Pa_ADN_ showed borderline values (0.78–0.80), with small differences from Pd/Pa_PAP_ (≤ 0.03).

### Anatomically non-significant vessels

Table 4 compares the patient and lesion characteristics in the vessels with DS ≤ 50% between the reverse mismatch (Pd/Pa_ADN_ ≤ 0.80) and non-reverse mismatch (Pd/Pa_ADN_ > 0.80) groups. The reverse mismatch group was associated with male sex (82% vs. 67%, *p* < 0.039), a higher frequency of non-LAD location (72% vs. 54%, *p* = 0.021), a smaller reference diameter (2.6 mm [IQR 2.3–3.1 mm]) vs. 2.9 mm [IQR 2.6–3.3 mm], *p* = 0.002), and a smaller minimal luminal diameter (1.5 mm [IQR 1.3–1.8 mm]) vs. 1.7 mm [IQR 1.6–2.1 mm], *p* < 0.001) compared to the non-reverse mismatch group. No significant difference in the overestimation of FFR was observed between the two groups (*p* = 0.224).

**Table 4:** Comparion clinical and lesion characteristics in vessels with DS ≤ 50% between the anatomical–functional reverse mismatch and non-reverse mismatch groups.

	Reverse mismatch Pd/Pa_ADN_ ≤ 0.80 (n = 76)	Non-mismatch Pd/Pa_ADN_ > 0.80 (n = 79)	*p* value
Age, yrs	71 (63–78)	72 (67–78)	0.436
Male, n (%)	62 (82%)	53 (67%)	0.039
Body mass index, kg/m^2^	24.6 (22.0–27.0)	23.4 (21.2–25.5)	0.055
Hypertension, n (%)	59 (78%)	61 (77%)	0.235
Diabetes mellitus, n (%)	32 (42%)	30 (38%)	0.600
Dyslipidemia, n (%)	46 (61%)	55 (70%)	0.779
Target vessel (LAD), n (%)	55 (72%)	43 (54%)	0.021
Proximal lesion*, n (%)	34 (45%)	30 (38%)	0.393
Multivessel disease, n (%)	44 (58%)	41 (52%)	0.453
**Quantitative coronary angiography**			
Reference diameter, mm	2.6 (2.3–3.1)	2.9 (2.6–3.3)	0.002
Minimal luminal diameter, mm	1.5 (1.3–1.8)	1.7 (1.6–2.1)	< 0.001
DS, %	42.5 (38.7–46.6)	41.5 (35.1–45.6)	0.058
Lesion length, mm	11.1 (7.6–17.0)	9.6 (7.2–15.0)	0.321
Hemodynamic parameters			
Heart rate at baseline, beats/min	65 (57–71)	67 (61–74)	0.102
Pa at baseline, mmHg	90 (79–98)	94 (83–100)	0.128
Pd/Pa ratio at baseline	0.91 (0.87–0.93)	0.95 (0.93–0.99)	< 0.001
Pd/Pa_ADN_	0.75 (0.69–0.78)	0.87 (0.84–0.91)	N.A
Pd/Pa_PAP_	0.73 (0.66–0.76)	0.86 (0.82–0.89)	N.A
Pd/Pa_ADN_ – Pd/Pa_PAP_	0.03 (0.01–0.03)	0.01 (0–0.04)	0.224

Values are expressed as medians (interquartile ranges) or frequencies (percentages). The reverse mismatch was defined as DS ≤ 50% and Pd/Pa_ADN_ ≤ 0.80, and the non-reverse mismatch as DS ≤ 50% and Pd/Pa_ADN_ > 0.80.

*Proximal location was defined as Syntax segments 1, 5, 6, and 11.

*DS* diameter stenosis, *LAD* left anterior descending coronary artery, *Pa* aortic pressure, *Pd/Pa* distal-to-aortic pressure ratio, *Pd/Pa*_*ADN*_ distal-to-aortic pressure ratio associated with adenosine, *Pd/Pa*_*ADN*_ distal-to-aortic pressure ratio associated with papaverine.

Based on the results of the univariable analysis (Suppl. Table S2), male sex (*p* = 0.042), body mass index (*p* = 0.061), LAD location (*p* = 0.022), reference diameter (*p* = 0.001), and minimal luminal diameter (*p* < 0.001) were included in the multivariable model. The results of the multivariable logistic regression analysis revealed the following as independent factors associated with the reverse mismatch (Table 5): male sex (OR: 2.43 [95% CI: 1.07–5.51], *p* = 0.034), LAD location (OR: 2.22 [95% CI: 1.05–4.67], *p* = 0.037), and minimal luminal diameter (OR per 0.1 mm increase: 0.75 [95% CI: 0.61–0.91], *p* = 0.034) as independent factors associated with the reverse mismatch (Table 5).

**Table 5:** Association with anatomical-functional reverse mismatch in multivariable analysis.

	OR	95% CI	*p* value
Male sex	2.43	1.07–5.51	0.034
Body mass index (per 1 kg/m^2^ increase)	1.06	0.96–1.18	0.233
LAD location	2.22	1.05–4.67	0.037
Reference diameter (per 0.1 mm increase)	3.69	0.92–1.19	0.498
Minimal luminal diameter (per 0.1 mm increase)	0.75	0.61–0.90	0.005

*CI* indicates confidence interval, *LAD* left anterior descending coronary artery, *OR* odds ratio.

Compared to adenosine, papaverine significantly increased the number of vessels with the anatomical–functional reverse mismatch (76 [49%] vs. 85 [55%], *p* = 0.022). In the non-reverse mismatch group, reclassification of functional significance was observed in 11 vessels (14%). In the reverse mismatch group, reverse reclassification was in observed only 2 vessels (3%).

## Discussion

Evaluating FFR values obtained with adenosine and papaverine in relation to DS on coronary angiography, we found that 1) the overestimation of FFR by adenosine was an independent determinant of the anatomical–functional mismatch; 2) in 30% of vessels with the anatomical–functional mismatch, functional significance was reclassified from negative by adenosine (Pd/Pa_ADN_ > 0.80) to positive by papaverine (Pd/Pa_PAP_ ≤ 0.80); and 3) this reclassification was observed more frequently in LAD lesions (52%) than in non-LAD lesions (20%).

### Anatomical–functional mismatch and overestimation of FFR by adenosine

An animal experimental study published in 1974 showed that hyperemic coronary flow starts to decrease from a DS of 50%^27^. Based on this result, the 50% threshold continues to be used to define anatomically significant stenosis. Obviously, a result derived from a controlled experiment in healthy animals cannot be accurately applied to patients with coronary artery disease. Patients requiring an FFR assessment often have diffuse atherosclerosis other than that at the site with minimal lumen diameter, which progressively decreases coronary pressure^28,29^. Unlike two-dimensional angiographic projection images, FFR reflects the totality of the physiological effects of patient characteristics, lesion characteristics, microvascular function, amount of myocardial mass subtended by the coronary artery stenosis, and hyperemia^3,7,11,12,30–32^. Consistent with prior studies^3,12^, non-LAD location and minimal luminal diameter were associated with an anatomical–functional mismatch. This study is first to demonstrate that the overestimation of FFR by adenosine is an independent determinant after adjusting for these known factors in the multivariable model.

There could be several potential factors associated with the overestimation of FFR observed in the present study. Although the 140 μg/kg/min dose of intravenous adenosine used in the present study has been recognized as the standard method for hyperemia induction^9,13,15^, a higher dose may be required, specifically when administered through a peripheral vein^33^.

Another potential explanation would be caffeine. The effects of caffeine on inhibiting adenosine hyperemia are definitively established^34^.While individually variable, caffeine reduces the average accuracy of all diagnostic metrics using vasodilatory stress. In the present study of Pd/Pa_PAP_ compared to Pd/Pa_ADN_ without systematic pre-procedure instruction for caffeine abstinence, higher Pd/Pa_ADN_ values than Pd/Pa_PAP_ values may have largely reflected caffeine effects in addition to other primary pharmacologic differences. Although a non-invasive imaging guideline recommends 12-h caffeine abstinence before adenosine stress tests^35^, more prolonged abstinence may be required for adenosine-induced FFR measurements. The overestimation of FFR by adenosine occurred at much lower serum caffeine levels than those found after the recommended 12-h abstinence and increased in a concentration–response manner^19^. Our present findings mirrored real-world clinical situations. The frequency of anatomical–functional mismatch was consistent with that of earlier investigations that used adenosine or adenosine triphosphate^3,12^. Although those investigations did not provide information about serum caffeine levels or the length of caffeine abstinence^3,12^, their results might be attributed in part to caffeine. Our present results suggest the need for standardized caffeine control before FFR measurements.

### Reclassification of functional significance due to FFR_ADN_ overestimation

A large randomized trial confirmed that revascularization reduced adverse cardiac events in patients who had coronary stenosis with FFR ≤ 0.80^10^. Recent guidelines consider coronary lesions with DS > 50% and FFR ≤ 0.80 to be indicated for revascularization^8,36^. In this study conducted on real-world patients, the functional significance was reclassified from negative by adenosine to positive by papaverine in as many as 30% of vessels with the anatomical-functional mismatch. Notably, more than half of vessels with reclassification status had a large overestimation of FFR by adenosine more than 2 standard deviations between repeated adenosine-induced FFR measurements^24^. Even FFR values in the gray zone (0.76–0.80) were associated with a high rate of death or myocardial infarction when treated with medical therapy alone^37^. The reclassification, specifically due to a large overestimation of FFR, may lead to worse clinical outcomes.

The present and previous studies found non-LAD location to be an independent determinant of the anatomical–functional mismatch^3,12^. This is a reasonable result from a physiological point of view. Stenosis in the non-LAD vessels, which subtends smaller myocardial masses than that in the LAD, produces a less hyperemic gradient across the lesion. Even with the anatomically significant stenosis of DS > 50%, the non-LAD is less likely to have FFR ≤ 0.80 than the LAD. Indeed, in the present study, the frequency of the anatomical–functional mismatch was higher in the non-LAD than in the LAD. Of note, however, the reclassification was observed in more than one-half of LAD vessels with the anatomical–functional mismatch. Physicians should keep in mind that when using adenosine, the anatomical–functional mismatch, specifically in the LAD, may suggest a false-negative result.

### Factors associated with anatomical–functional reverse mismatch

In agreement with previous studies^3,5,12^, our analyses revealed that male sex, LAD location, and minimal lumen diameter are independent determinants of the anatomical–functional reverse mismatch. A greater pressure gradient is produced across a lesion located in the LAD, subtending larger myocardial masses than a lesion located in the non-LAD^3,12^. Likewise, males (with larger myocardial masses than females) are likely to have lower FFR values^5^.

### Clinical implications

Given that revascularization reduces adverse cardiac events even in patients with gray zone FFR values compared with medical therapy alone^38^, patients with overestimations of FFR may miss the opportunity to receive the benefits of revascularization. The concept of FFR was established under the assumption of minimal microvascular resistance^9^. Our findings underscore the importance of inducing maximal hyperemia in FFR measurements to avoid misinterpretations of the FFR results. Significantly, more than half of LAD lesions with the anatomical–functional mismatch demonstrated false-negative results. Considering that stenotic lesions in the LAD carry a worse prognosis than in other locations^39^, LAD lesions with anatomical–functional mismatch are more clinically relevant.

FFR overestimations by adenosine (i.e. insufficient adenosine-induced hyperemia) will not be identified unless another hyperemic stimulus is used. Serum caffeine was reported to be a major determinant^19^; however the serum caffeine level is unpredictable even after caffeine abstinence because it depends on various factors, including the inter-individual variation in caffeine’s half-life, patient compliance, and the recently ingested caffeine dose. In the context of a lack of a rapid test kit to detect serum caffeine, the anatomical–functional mismatch provides a clue to insufficient adenosine-induced hyperemia.

### Study limitations

We acknowledge several limitations to this study. First, we did not determine the clinical significance of the anatomical–functional mismatch and/or the reclassification due to the overestimation of FFR by adenosine. This was because some of the vessels with DS > 50% and negative FFR results by adenosine were revascularized based on positive FFR results by papaverine. Further research is warranted to address this topic. Second, microvascular function was not assessed. High microcirculatory resistance might affect the overestimation of FFR. However, in the presence of insufficient adenosine-induced hyperemia, microcirculatory resistance cannot be accurately assessed by adenosine. Multiple injections of papaverine to measure microcirculatory resistance, together with FFR, may carry an increased risk of side effects. Third, the patients' serum caffeine levels were not measured in this study. Lastly, the order of hyperemic agents was fixed (papaverine last) because papaverine was used to obtain a reliable pullback curve. Although papaverine was administered after confirming that Pd/Pa values had returned to the baseline level, adenosine’s carry-over effect cannot be excluded.

## Conclusions

The overestimation of FFR by adenosine compared to papaverine is an independent determinant of anatomical–fucntional mismatch. It should be kept in mind that anatomical–functional mismatch, specifically in the LAD and potentially due to caffeine, may suggest a false-negative result.

## Supplementary Information

Supplementary Information.

## Abbreviations

CI: Confidence interval
DS: Diameter stenosis
Pd/Pa: Distal-to-aortic pressure ratio
Pd/Pa_ADN_: Distal-to-aortic pressure ratio associated with adenosine
Pd/Pa_PAP_: Distal-to-aortic pressure ratio associated with papaverine
FFR: Fractional flow reserve
IQR: Interquartile range
LAD: Left anterior descending artery
OR: Odds ratio
Pa: Mean aortic pressure
Pd: Mean distal coronary pressure
