# Supplementary material for: Impact of overestimation of fractional flow reserve by adenosine on anatomical–functional mismatch
Source: Sci Rep. 2022 Sep 2;12:14962. doi: 10.1038/s41598-022-19330-1 (PMC9440099; doi:10.1038/s41598-022-19330-1)
Supplement: Supplementary file 1 — Supplementary Information. [file 41598_2022_19330_MOESM1_ESM.docx]

**Supplementary material**

**Title:** Impact of overestimation of fractional flow reserve by adenosine on anatomical–functional mismatch

**Authors:** Hidenari Matsumoto, Ryota Masaki, Satoshi Higuchi, Hideaki Tanaka, Seita Kondo, Hiroaki Tsujita, and Toshiro Shinke

**Table S1. Association with anatomical-functional mismatch in univariable analysis**

|  | OR | 95% CI | p value |
| --- | --- | --- | --- |
| Age (per 1 year increase) | 1.01 | 0.98–1.05 | 0.388 |
| Female sex | 1.49 | 0.70–3.17 | 0.299 |
| Body mass index (per 1 kg/m^2^ increase) | 0.93 | 0.85–1.02 | 0.128 |
| Hypertension | 0.98 | 0.52–1.86 | 0.959 |
| Diabetes mellitus | 0.92 | 0.49–1.70 | 0.779 |
| Dyslipidemia, n (%) | 1.25 | 0.59–2.65 | 0.553 |
| Non-LAD location | 6.48 | 3.30–12.70 | <0.001 |
| Non-proximal lesion* | 1.74 | 0.90–3.37 | 0.102 |
| Multivessel disease | 1.15 | 0.62–2.12 | 0.658 |
| Quantitative coronary angiography |  |  |  |
| Reference diameter (per 0.1 mm increase) | 1.08 | 1.02–1.13 | 0.004 |
| Minimal luminal diameter (per 0.1 mm increase) | 1.19 | 1.08–1.31 | 0.001 |
| Lesion length (per 1 mm increase) | 0.98 | 0.95–1.01 | 0.250 |
| Hemodynamic parameters |  |  |  |
| Heart rate at baseline (per 1 beat/min increase) | 1.00 | 0.97–1.02 | 0.748 |
| Pa at baseline (per 1 mmHg increase) | 1.01 | 0.99–1.03 | 0.213 |
| Pd/Pa_ADN_ – Pd/Pa_PAP_ (per 0.01 increase) | 1.11 | 1.02–1.20 | 0.014 |

Values are expressed as medians (interquartile ranges) or frequencies (percentages).

^*^Proximal location was defined as Syntax segments 1, 5, 6, and 11.

DS indicates diameter stenosis; LAD, left anterior descending coronary artery; CI, confidence interval; OR, odds ratio; Pa, mean aortic pressure; Pd/Pa_ADN_, distal-to-aortic pressure ratio associated with adenosine; and Pd/Pa_PAP_, distal-to-aortic pressure ratio associated with papaverine.

**Table S2. Association with anatomical-functional reverse mismatch in univariable analysis**

|  | OR | 95% CI | p value |
| --- | --- | --- | --- |
| Age (per 1 year increase) | 0.99 | 0.95–1.02 | 0.392 |
| Male sex | 2.17 | 0.02–0.77 | 0.042 |
| Body mass index (per 1 kg/m^2^ increase) | 1.09 | 1.00–1.20 | 0.061 |
| Hypertension | 1.02 | 0.48–2.18 | 0.951 |
| Diabetes mellitus | 1.19 | 0.62–2.26 | 0.600 |
| Dyslipidemia, n (%) | 0.67 | 0.34–1.30 | 0.236 |
| LAD location | 2.19 | 1.12–4.28 | 0.022 |
| Proximal lesion* | 1.32 | 0.70–2.51 | 0.393 |
| Multivessel disease | 1.27 | 0.68–2.40 | 0.454 |
| Quantitative coronary angiography |  |  |  |
| Reference diameter (per 0.1 mm increase) | 0.90 | 0.84–0.96 | 0.001 |
| Minimal luminal diameter (per 0.1 mm increase) | 0.80 | 0.72–0.88 | <0.001 |
| Lesion length (per 1 mm increase) | 1.03 | 0.99–1.07 | 0.175 |
| Hemodynamic parameters |  |  |  |
| Heart rate at baseline (per 1 beat/min increase) | 0.98 | 0.95–1.00 | 0.103 |
| Pa at baseline (per 1 mmHg increase) | 0.99 | 0.97–1.01 | 0.288 |
| Pd/Pa_ADN_ – Pd/Pa_PAP_ (per 0.01 increase) | 1.00 | 0.90–1.10 | 0.928 |

Values are expressed as medians (interquartile ranges) or frequencies (percentages).

^*^Proximal location was defined as Syntax segments 1, 5, 6, and 11.

DS indicates diameter stenosis; LAD, left anterior descending coronary artery; CI, confidence interval; OR, odds ratio; Pa, mean aortic pressure; Pd/Pa_ADN_, distal-to-aortic pressure ratio associated with adenosine; and Pd/Pa_PAP_, distal-to-aortic pressure ratio associated with papaverine.
